# Kinetics and Mechanism of Cation‐Induced Guest Release from Cucurbit[7]uril

**DOI:** 10.1002/chem.201905633

**Published:** 2020-05-19

**Authors:** Zsombor Miskolczy, Mónika Megyesi, László Biczók, Amrutha Prabodh, Frank Biedermann

**Affiliations:** ^1^ Institute of Materials and Environmental Chemistry Research Centre for Natural Sciences P.O. Box 286 1519 Budapest Hungary; ^2^ Institute of Nanotechnology (INT) Karlsruhe Institute of Technology (KIT) Hermann-von-Helmholtz-Platz 1 76344 Eggenstein-Leopoldshafen Germany

**Keywords:** host–guest systems, kinetics, reaction mechanisms, salt effect, self-assembly

## Abstract

The release of two organic guests from cucurbit[7]uril (CB7) was selectively monitored by the stopped‐flow method in aqueous solutions of inorganic salts to reveal the mechanistic picture in detail. Two contrasting mechanisms were identified: The symmetric dicationic 2,7‐dimethyldiazapyrenium shows a cation‐independent complex dissociation mechanism coupled to deceleration of the ingression in the presence of alkali and alkaline earth cations (M^*n*+^) due to competitive formation of CB7–M^*n*+^ complexes. A much richer, unprecedented kinetic behaviour was observed for the ingression and egression of the monocationic and non‐symmetric berberine (B^+^). The formation of ternary complex B^+^–CB7–M^*n*+^ was unambiguously revealed. A difference of more than two orders of magnitude was found in the equilibrium constants of M^*n*+^ binding to B^+^–CB7 inclusion complex. Large cations, such as K^+^ and Ba^2+^, also promoted B^+^ expulsion from the ternary complex in a bimolecular process. This study reveals a previously hidden mechanistic picture and motivates systematic kinetic investigations of other host–guest systems.

## Graphical abstract

**Contrasting mechanisms**: Stopped‐flow studies on the release of two organic guests from cucurbit[7]uril (CB7) in aqueous solutions of inorganic salts revealed two contrasting mechanisms. Whereas metal cations M^*n*+^ promote the release of berberine (B^+^) from CB7 in a multistep process, the expulsion of dicationic dye 2,7‐dimethyldiazapyrenium has a simpler mechanism.

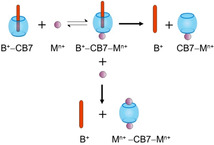

## Introduction

The rigid, biocompatible cucurbituril (CB*n*, *n=*5–8) macrocycles are widely used building blocks in supramolecular chemistry and nanotechnology.^1^ They have a continuously expanding range of applications in the biomedicine,^2^ drug delivery,^3^ catalysis^4^ and sensing.^5^ The nonpolar, low‐polarisability CB*n* cavity readily includes hydrophobic moieties, whereas the high electron density of the carbonyl‐laced portals facilitates the interaction with cations. The electrostatic and hydrophobic effects combined with the complementary dimensions of CB*n* interior and guests leads to particularly strong binding of cationic organic compounds.^6^ Metal cations (M^*n*+^) are readily coordinated by the oxygen atoms of the host entrance with higher binding affinity to CB*n* than to the well‐known classical cation receptor 18‐crown‐6.^7^ The cooperative binding of several Ca^2+^ or Na^+^ ions to thioflavin–(CB7)_2_ complex was found to produce highly fluorescent supramolecular nanocapsules,^8^ whereas the coordination of transition‐metal ions to the rim of CB7 altered the photodeazetation of encapsulated bicyclic azoalkane guests.^9^ Na^+^ addition can be used to induce the transfer of neutral red dye from the CB7 cavity to the pocket of bovine serum albumin.^10^

Kinetic data on guest capture and release are essential for many applications of CB*n* complexes,^11^ including the rational design of molecular switches,^12^ self‐sorting systems^13^ and light‐driven control of supramolecular assemblies.^14^ Time‐resolved NMR studies demonstrated that the experimentally measured rate constant for ingression of the cyclohexylmethylammonium ion into CB6 substantially diminished with increasing Na^+^ concentration, whereas the egression rate constant barely changed.^15^ The increase in cation size caused an approximately twofold increase in the ingression rate constant, but the egression rate constant remained essentially constant for such CB6 complexes.^15^ Competitive binding of Na^+^ decelerated the inclusion of organic guests in CB7 into the time range of the stopped‐flow technique.^16^ The formation of Na^+^–CB7 and Na^+^–CB7–Na^+^ complexes lessened the concentration of free CB7 and thereby slowed down the bimolecular ingression. Systematic time‐resolved studies on 2‐naphthyl‐1‐ethylammonium encapsulation demonstrated that this guest neither expelled Na^+^ nor produced a ternary complex in the reactions with Na^+^–CB7.^17^ A ditopic guest, namely the *N*‐phenyl‐2‐naphthylammonium cation, produced two types of 1:1 complex with CB7. When the phenyl group was embedded in the macrocycle, the binding of Na^+^ to the complex slowed down the guest release. In contrast, Na^+^ sped up the exit of the guest through competitive expulsion when the naphthyl moiety was confined.^18^ Such investigations are possible if the association of M^*n*+^ with guest–CB*n* complexes leads to changes in the luminescence characteristics, as was observed in several instances.^19^

**Scheme 1:**
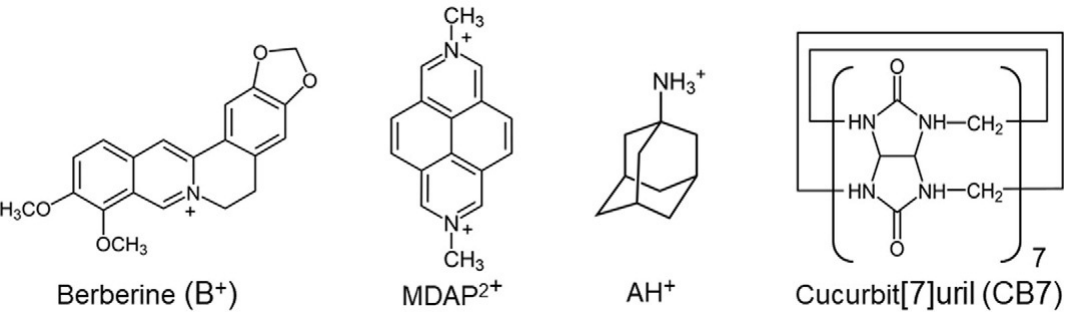
Chemical structures of the guest and host compounds.

It is known for CB*n* host–guest complexes that the apparent binding constant of guest encapsulation is reduced in the presence of inorganic salts,5c, 15, 20 but it is unknown to what extent this effect arises from competitive binding of M^*n*+^ to form CB*n*–M^*n*+^ complexes or from the formation of less‐stable guest–CB*n*–M^*n*+^ ternary complexes. Although it is difficult to obtain such mechanistic insights by affinity measurements, we show here that these two scenarios can be easily distinguished by kinetic studies. However, it is not yet fully understood how the various metal cations influence the kinetics and mechanism of guest exit from the CB*n* cavity. Specifically, the monitoring of the decomplexation kinetics will give the deepest insight into the subtle details of the mechanism of guest release in salt solutions: In a competitive binding mechanism, the rate of host–guest complex dissociation should be independent of the cation concentration, whereas the guest egression rate from a guest–CB*n*–M^*n*+^ ternary complex will be cation‐dependent. In reality, it can be expected that the competitive binding mechanism is always present. The practical challenging task is therefore to evaluate whether or not this is complemented by a simultaneously present mechanism in which guest release occurs from a guest–CB*n*–M^*n*+^ ternary complex. The commonly applied kinetic analysis procedure—fitting of the kinetic traces for host‐guest association recorded after mixing of host and guest solutions^11^, ^21^—provides consistent but not sufficiently accurate results, because three unknown parameters (i.e., a “signal parameter” for converting the concentration to fluorescence intensity, rate constants of ingression and egression) must be optimised. In addition, the rate of bimolecular complexation reactions is influenced by the initial concentrations of the various species produced in the solution, which can be calculated only if the association constants and the binding mechanism are known. The interpretation of the salt effect on the unimolecular complex dissociation is much simpler and permits selective measurement of the rate constant of guest release *k*_out_ with high accuracy, as we have shown recently.^22^ In essence, a strongly binding organic competitor is added in excess to a solution of the host and guest to trigger complex dissociation of the host–guest complex according to a pseudo‐first order kinetic path. Notably, unknown concentrations of the individual species/complexes initially present, that is, CB7, CB7–M^*n*+^, M^*n*+^–CB7–M^*n*+^ and guest–CB7 do not influence *k*_out_ and thus permit the determination of *k*_out_ with the required high accuracy. In this study, we utilised the *k*_out_ method to investigate systematically the effects of inorganic salts on the competitive versus ternary complex formation/dissociation kinetics and mechanism of host–guest complexation with CB7. The investigations revealed the effect of the size and charge of M^*n*+^ on the rate and the importance of each dissociation step. In addition, we unravelled whether increasing the positive charge of the encapsulated molecule alters the dynamics of decomplexation. Berberine (B^+^), a singly charged pharmaceutically important isoquinoline alkaloid, and 2,7‐dimethyldiazapyrenium (MDAP^2+^) dicationic dye were chosen as guest compounds because of their high binding affinity and the considerable alteration of their fluorescent behaviour upon confinement in CB7.19c, 23 Scheme 1 presents the structural formula of the utilised compounds.

## Results

### MDAP^2+^ exit from its CB7 complex is independent of the type and concentration of cations

As a first example, the dicationic and symmetric MDAP^2+^ was used as a guest for CB7. It is unlikely that formation of ternary complex MDAP^2+^–CB7–M^*n*+^ can occur, because the positively charged ends (i.e., *N*‐CH_3_ groups) of this guest are symmetrically placed at the portals of CB7 in the MDAP^2+^–CB7 complex,^23^ which should prevent simultaneous association of a metal cation M^*n*+^ with the CB7 portal. Therefore, the system of MDAP^2+^ and CB7 can be expected to only show the hallmarks of the competitive, salt‐induced complex‐dissociation mechanism, which makes it a suitable, simpler starting point. The marked alteration of its absorption and fluorescence spectra upon addition of CB7 (Figure S1 in the Supporting Information) implied formation of an inclusion complex. The significantly weaker emission of MDAP^2+^ at 454 nm when excited at 339 nm in water than in the cavity of CB7 was exploited to selectively detect complex dissociation by mixing equimolar solutions of MDAP^2+^ and CB7 (5 μm at *t*=0 s) with a solution of 1‐adamantylammonium cation (AH^+^, 300 μm at *t*=0 s). The exponential fluorescence‐intensity decay (Figure S2 a in the Supporting Information) was rather slow; a nonlinear least‐squares fit provided *k*_out_=0.015±0.001 s^−1^ for the rate constant of MDAP^2+^ egression from the cavity of CB7 in water.

**Scheme 2:**
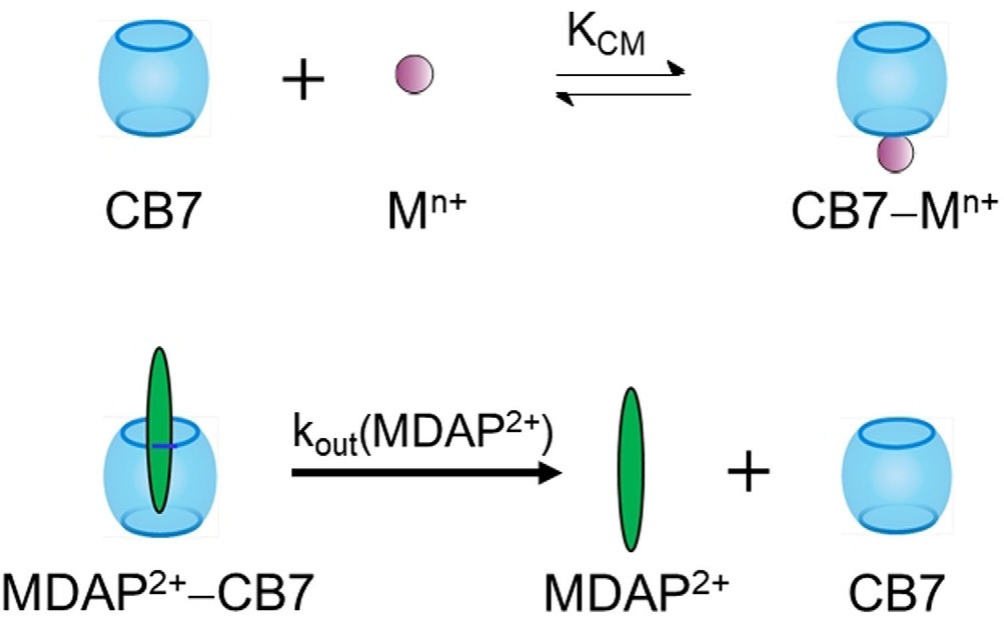
Mechanism of MDAP^2+^ release from CB7.

The experiments were repeated at various Ca(NO_3_)_2_ or Ba(NO_3_)_2_ concentrations. As seen in Table 1, the obtained *k*_out_ values barely varied with the concentration and type of salt. In explorative experiments at high concentrations (140 mm), Li^+^ and Na^+^ salts showed similar results, that is, salt‐independent *k*_out_ values for release of MDAP^2+^ from the MDAP^2+^–CB7 complex. Thus, it can be concluded that inorganic cations have negligible interaction with the MDAP^2+^–CB7 inclusion complex, that is, as expected, ternary‐complex formation does not occur. Hence, MDAP^2+^ dissociation can be modelled by the simple reaction steps presented in Scheme 2.

**Table 1:** Effect of Ca^2+^ and Ba^2+^ concentrations on the rate constant of MDAP^2+^ exit from CB7.

Ca^2+^ [mm]	*k*_out_^[a]^ [s^−1^]	Ba^2+^ [mm]	*k*_out_^[a]^ [s^−1^]
0	0.0153	0	0.0153
0.5	0.0159	0.5	0.0172
1.0	0.0171	1.0	0.0175
3.5	0.0179	2.0	0.0176
5.0	0.0175	3.5	0.0177
10.0	0.0181	5.0	0.0180
25.0	0.0186	10.0	0.0192

[a] Estimated error is ±6 %.

The slow MDAP^2+^ egression probably arises from the substantial activation enthalpy of the process. The guests bearing double positive charges are usually more strongly bound in CB7 than the singly charged ones because of their enhanced electrostatic interactions with the electron‐rich oxygen atoms at the portals of the host.1c, 1d, 24 In addition, the passage of large MDAP^2+^ through the CB7 portal is expected to be sterically hindered.^23^ In general, we believe that salt‐insensitive dissociation kinetics are expected for doubly charged guests with symmetric charge localisation near the CB*n* portals, while for singly or uncharged guests, different mechanisms (e.g., ternary‐complex formation; see below for berberine) may occur depending on the type and concentration of inorganic cations present.

### B^+^ exit from its CB7 complex is strongly dependent on the type and concentration of cations

**Figure 1:**
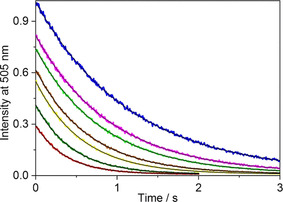
Stopped‐flow signals at 505 nm in a solution of B^+^ and CB7 after mixing with AH^+^ solution in the presence of 0, 0.32, 0.53, 1.0, 1.6, 2.7 and 4.4 mm CaCl_2_ (from top to bottom). Total concentrations at *t*=0 s were [B^+^]_T_=[CB7]_T_=0.25 μm and [AH^+^]_T_=5 μm. Excitation occurred at 345 nm. The black lines represent the result of the nonlinear least‐squares analysis.

**Figure 2:**
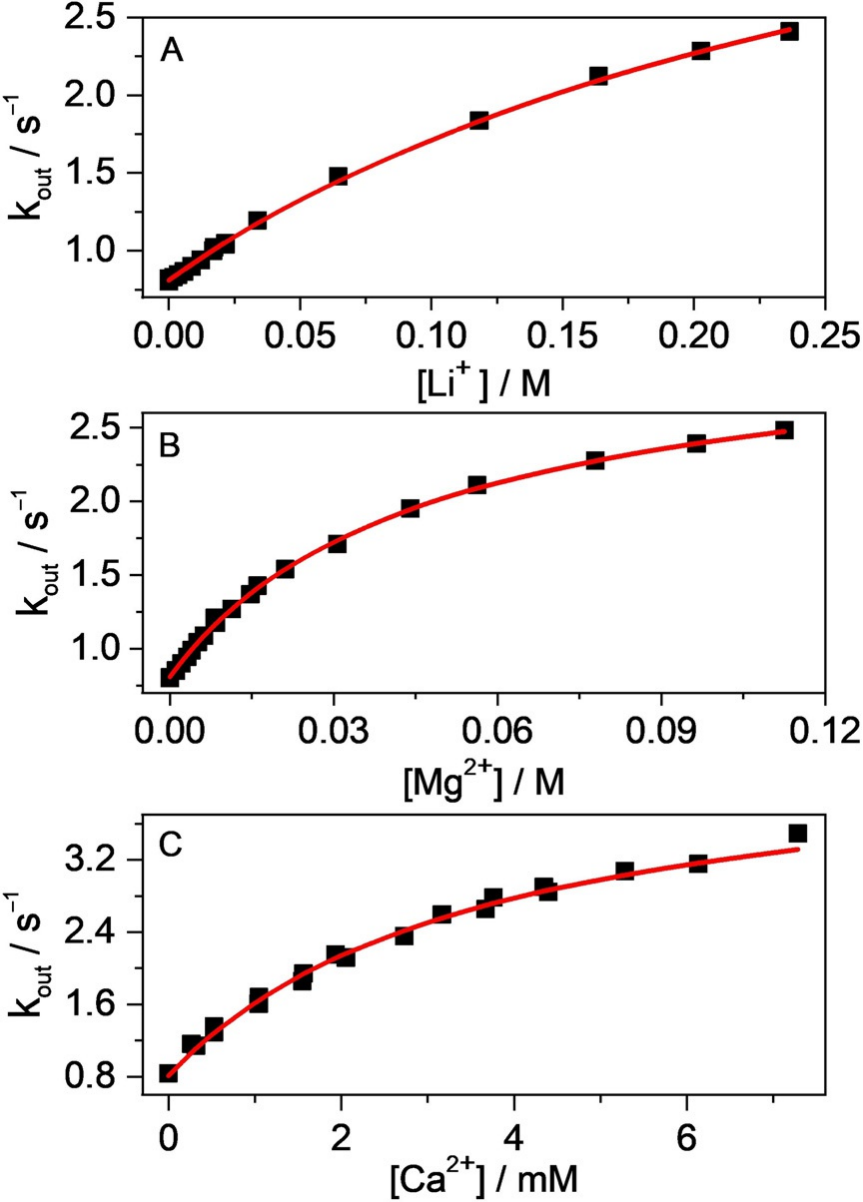
Effect of A) LiCl, B) MgSO_4_ and C) CaCl_2_ concentrations on the apparent rate constant of B^+^ release from CB7. Lines represent the fitted function.

In contrast to MDAP^2+^, the alkaloid berberine (B^+^) is singly positively charged with a charge delocalisation near one end of the molecule. Besides, the calculated complex structure with CB7 indicates a highly non‐symmetric complex geometry, which suggests that one CB7 portal area may be available for simultaneous M^*n*+^ binding.19c B^+^ is a particularly advantageous guest compound for mechanistic studies because it has negligible emission in water but is highly fluorescent in the cavity of CB7.19c Hence, the variation of its fluorescence intensity directly reflects changes in the concentration of CB7‐bound B^+^. To gain insight into the reaction steps leading to inclusion‐complex dissociation in the presence of salts, we selectively monitored B^+^ egression from CB7 by the *k*_out_ method.^22^ As a representative example, Figure 1 shows the fluorescence intensity decrease at 505 nm in the equimolar solution of B^+^ and CB7 after mixing with AH^+^ solution in the presence of various CaCl_2_ concentrations. Because of the dilution, a fraction of B^+^–CB7 complex dissociated and AH^+^ quickly occupied the cavity of free CB7. Thereby, the back‐formation of B^+^–CB7 was essentially irreversibly blocked and the exponential fluorescence intensity decays showed re‐establishment of the equilibrium by diminution of the B^+^–CB7 concentration. The fit of the stopped‐flow traces (Figure 1) with an exponential function provided the apparent rate constants of B^+^ exit from CB7 (*k*_out_). When the amount of Ca^2+^ was increased, the incipient fluorescence intensity decreased because the competitive association of Ca^2+^ with CB7 interfered with B^+^ inclusion. A similar effect was found in the presence of Li^+^ and Mg^2+^ cations. Figure 2 shows that *k*_out_ increases and reaches a plateau at high M^*n*+^ concentrations. This phenomenon is due to the association of cations with the B^+^–CB7 complex. Subsequently, B^+^ dissociates faster from the produced ternary complex (B^+^–CB7–M^*n*+^). As the fraction of B^+^‐CB7‐M^*n*+^ grows, the apparent rate constants gradually increase and the changes level off at high cation concentrations at which the ternary complex dominates.

To uncover whether also the type of anion affects the kinetics and mechanism of B^+^ dissociation from CB7, measurements were carried out in various potassium salt solutions. Changing the anion modified the kinetic traces only to a negligible extent, and the calculated parameters agreed within the limit of experimental errors (Table 2). This observation also indicates that ionic strength does not influence B^+^ release, because otherwise different results would be expected in the solution containing doubly charged SO_4_^2−^ than in the presence of singly charged Cl^−^ and NO_3_^−^ anions.

**Table 2:** Variation of the calculated parameters with the radius and charge of metal cations.

Metal salt	M^*n*+^ radius [pm]^[a]^	*K*_CM_ [m ^−1^]	*β*/*α*	*K*_BCM_ [m ^−1^]	*k*_out_(BCM) [s^−1^]	*k*_out_(BCMM) [m ^−1^ s^−1^]
LiCl	69	220^[b]^	0.67±0.05	5±3	4.4±0.4	–^[e]^
MgSO_4_	72	1740^[b]^	0.70±0.05	31±4	3.2±0.4	–^[e]^
CaCl_2_	100	14 000^[c]^	0.71±0.05	390±30	4.6±0.4	–^[e]^
KCl	138	2400^[c]^	0.66±0.05	83±5	5.0±0.4	27±4
K_2_SO_4_	138	2400^[c]^	0.66±0.05	81±5	5.0±0.4	23±4
KNO_3_	138	2400^[c]^	0.66±0.05	79±5	5.2±0.4	31±4
BaCl_2_	136	60 300^[d]^	0.73±0.05	1000±100	5.9±0.4	600±70

[a] In water.^25^ [b] From fluorescence displacement titration.^7^ [c] Average of the results of fluorescence displacement titration and isothermal titration calorimetry experiments.^7^ [d] By isothermal titration calorimetry.^7^ [e] This reaction step does not take place.

**Scheme 3:**
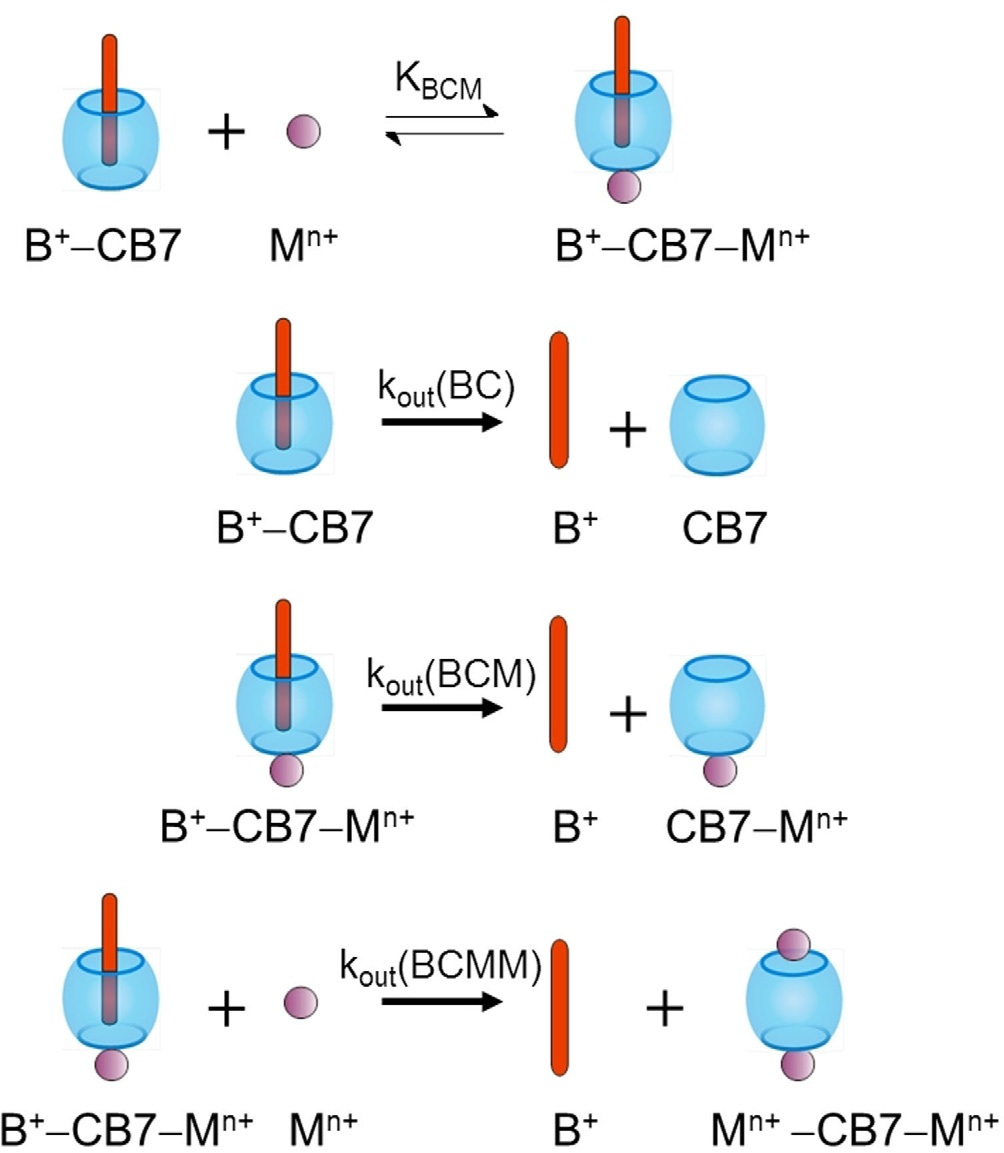
Mechanism of B^+^ release from the CB7 cavity.

In view of the cation‐dependent but anion‐independent dissociation kinetics, we therefore propose a composite mechanism of B^+^ release from its CB7 complex, that is, a combination of both of the usual competitive binding mechanisms (as also found for MDAP^2+^, see above) and the new ternary‐complex‐based dissociation pathway from the simultaneously present B^+^–CB7–M^*n*+^ complexes (Scheme 3).

For testing our mechanistic hypothesis, the equilibrium constant of B^+^–CB7–M^*n*+^ formation *K*_BCM_ and the rate constant of B^+^ exit from the ternary complex *k*_out_(BCM) were determined by fitting the experimental data to Equation (1), which models the cation‐dependent ternary‐complex‐based dissociation pathway (see the Supporting Information for the derivation of the mathematical expression).

(1) $$k_{\mathrm{out}}=k_{\mathrm{out}}\left(\mathrm{BC}\right)\frac{1}{1+\frac{\beta }{\alpha }K_{\mathrm{BCM}}\left[M^{n+}\right]}+k_{\mathrm{out}}\left(\mathrm{BCM}\right)\frac{\frac{\beta }{\alpha }K_{\mathrm{BCM}}\left[M^{n+}\right]}{1+\frac{\beta }{\alpha }K_{\mathrm{BCM}}\left[M^{n+}\right]}$$

where, *k*_out_(BC) is the rate constant of B^+^–CB7 dissociation measured in the absence of salts (0.81±0.08 s^−1^) and *β*/*α* the relative fluorescence yield of B^+^–CB7–M^*n*+^ and B^+^–CB7 at the detection wavelength (505 nm).

**Figure 3:**
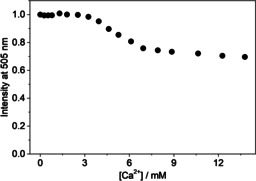
Fluorescence intensity as a function of Ca^2+^ concentration in a solution of 0.02 mm B^+^ and 1.0 mm CB7. Excitation was performed at 420 nm.

To determine the latter quantity, the fluorescence‐intensity variation was measured upon gradual addition of M^*n*+^ to B^+^–CB7 solution in steady‐state titrations. In these experiments, we used 0.020 mm B^+^ and 1 mm CB7 concentrations to ensure total complexation of B^+^ and to facilitate B^+^–CB7–M^*n*+^ formation. The samples were excited at 420 nm, at which the molar absorption coefficient of B^+^‐CB7 is relatively low (*ϵ*=5300 m ^−1^ cm^−1^), to prevent inner‐filter effects. As a typical example, Figure 3 shows the fluorescence intensities at various Ca^2+^ concentrations. Initially, the intensity remains constant because Ca^2+^ has moderate binding affinity to B^+^–CB7. The intensity decrease above 3 mm Ca^2+^ concentration is attributed to transformation of B^+^–CB7 into the more weakly emitting B^+^–CB7–Ca^2+^. Above approximately 6 mm Ca^2+^, that is, when B^+^–CB7 is fully converted to the more weakly emitting B^+^–CB7–Ca^2+^, the fluorescence intensity levels off and the emission is assigned to B^+^–CB7–Ca^2+^. The 50‐fold larger total amount of CB7 compared with that of B^+^ guarantees that free CB7 remains in large excess and, as a consequence, the concomitant formation of CB7–Ca^2+^ complex does not induce B^+^ release in the applied Ca^2+^ concentration range. Indeed, computer modelling calculations showed (Table S1 in the Supporting Information) that competitive binding of Ca^2+^ to CB7 causes negligible change in B^+^ concentrations and less than 0.14 % of the total B^+^ amount is free under the conditions of our study. The ratio of the intensities at the plateau and the initial ranges of Figure 3 gives the *β*/*α* parameter of Equation (1). Other cations bring about similar behaviour to that shown in Figure 3, and the derived *β*/*α* values are practically constant within the limit of experimental errors (Table 2). The results shown in Figure 2 were analysed by using Equation (1) with *β*/*α* values. Table 2 summarises the calculated *K*_BCM_ and *k*_out_(BCM) parameters. The metal‐cation radii in water^25^ and the equilibrium constants of 1:1 binding of M^*n*+^ to CB7 *K*_CM_ are also included.

**Figure 4:**
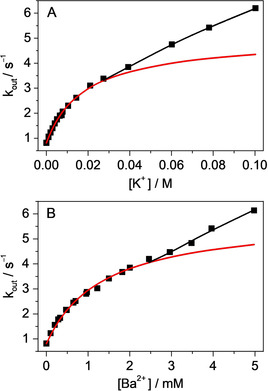
Apparent rate constant of B^+^ egression from CB7 as a function of A) K^+^ and B) Ba^2+^ concentration. Lines shows the fitted functions.

The mechanism observed with Li^+^, Mg^2+^ and Ca^2+^ cations also prevails at low K^+^ or Ba^2+^ concentrations when B^+^–CB7 dominates. The red lines in Figure 4 represent the results of the nonlinear least‐squares fit of Equation (1) to the experimental data measured at the low cation amounts. The calculated equilibrium constants for B^+^–CB7–M^*n*+^ formation *K*_BCM_ and the rate constants of the unimolecular dissociation of B^+^ from the ternary complexes *k*_out_(BCM) match the trend found with smaller cations (Table 2). The former quantity significantly varies, whereas less then twofold change is found in *k*_out_(BCM). Despite the similar radii of K^+^ and Ba^2+^, the latter ion produces a more stable ternary complex because of its higher positive charge.

Similarly, the affinity of CB*n*–M^*n*+^ complexes increases with increasing net charge of the cation due to increased ion–dipole interaction with the carbonyl‐fringed portals of the CB*n* macrocycles (see Table 2 and ref. 7) Likewise, despite the barely different sizes of Li^+^ and Mg^2+^, a much higher association strength *K*_BCM_ was found for the doubly‐charged Mg^2+^. The binding affinity to B^+^–CB7 complex considerably increases with increasing size of the cation in the series Mg^2+^<Ca^2+^<Ba^2+^. Such an effect also appears for K^+^ compared with Li^+^, for which more than one order of magnitude difference is found in *K*_BCM_.

Remarkably different, new kinetic features appeared at high K^+^ and Ba^2+^ concentrations when B^+^–CB7–M^*n*+^ outweighed B^+^–CB7 (Figure 4). Under these conditions, the apparent rate constant of B^+^ egression did not level off but linearly increased, which implies that large cations reacted with B^+^–CB7–M^*n*+^ in a bimolecular process. This is the first case in which such a bimolecular substitution mechanism was clearly demonstrated in the guest exchange of cucurbiturils. The motion of M^*n*+^ toward ternary complex B^+^–CB7–M^*n*+^ is probably coupled with the displacement of B^+^ from the host cavity and the production of M^*n*+^–CB7–M^*n*+^ complex. This reaction is akin to the S_E_2 type of electrophilic substitution in organic chemistry, in which the formation of the new bond and the breaking of the old bond take place simultaneously via a single transition state. The optimal size of M^*n*+^ is important, because cations with radius of approximately 100 pm or smaller appear unable to participate in such a process, whereas organic cations are too big and usually have delocalised charge, which makes this type of reaction unfavourable. From the linear contribution of the dependence of *k*_out_ on M^*n*+^ concentration, an approximately 20‐fold larger rate constant is derived for bimolecular B^+^ removal by Ba^2+^ than by K^+^ (*k*_out_(BCMM) in Table 2). Because of its double positive charge, the former ion more efficiently promotes the expulsion of B^+^.

**Figure 5:**
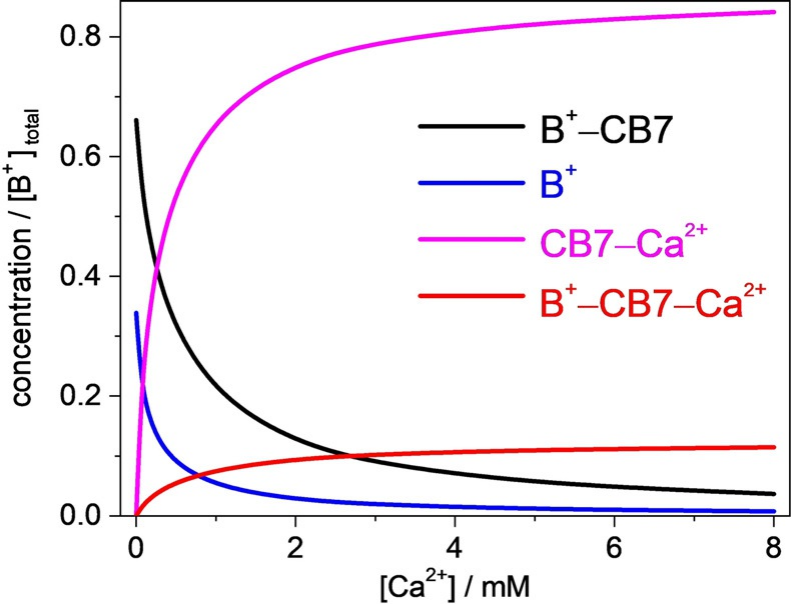
Composition of solutions at various Ca^2+^ concentrations and [B^+^]_total_=[CB7]_total_=0.25 μm.

**Figure 6:**
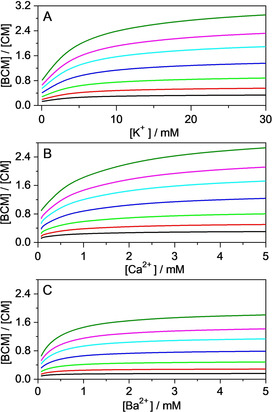
Variation of B^+^–CB7–M^*n*+^/ CB7–M^*n*+^ molar ratios in equilibrium as a function of A) K^+^, B) Ca^2+^ and C) Ba^2+^ concentrations in equimolar solutions of B^+^ and CB7. [B^+^]=[CB7]=15, 10, 7, 4, 2, 1, and 0.5 μm (from top to bottom).

To gain information on the relative importance of the dissociation pathways via ternary complex formation compared with the decomplexation due to the competitive binding of M^*n*+^ to CB7, we calculated how the concentration of the species participating in equilibria and the [B^+^–CB7–M^*n*+^]/ [CB7–M^*n*+^] molar ratio changes with the total amount of the constituents and the type of M^*n*+^. We focused on the salt concentration range in which M^*n*+^–CB7–M^*n*+^ formation does not play an important role. Derivation of the formulas is shown in the Supporting Information. The *K*_BCM_ values were taken from Table 2 and the previously published equilibrium constants were used for the association of metal cations^7^ and berberine^26^ with CB7. As a representative example, Figure 5 shows the calculated relationship between the concentration of the components and the total Ca^2+^ concentration in equimolar B^+^ and CB7 solution. The same total host and guest concentrations were employed as in the measurement of the data plotted in Figure 2 C ([B^+^]_total_=[CB7]_total_=0.25 μm). In the absence of salt, 66 % of the guest is complexed. When Ca^2+^ concentration is raised, the amount of CB7–M^*n*+^ steeply grows at the expense of B^+^ and B^+^–CB7. Less than 12 % of [B^+^]_total_ is converted to B^+^–CB7–Ca^2+^ in such dilute solution. As a measure of the relative importance of ternary‐complex formation compared with competitive association with CB7, we chose the ratio of B^+^–CB7–M^*n*+^ and CB7–M^*n*+^ concentrations ([BCM]/[CM]). Figure 6 shows this quantity as a function of M^*n*+^ concentration in equimolar B^+^ and CB7 solutions. As expected, [BCM]/[CM] considerably grows with increasing total concentration of the constituents, but the smallest increase appears in Ba^2+^ solutions because the affinities of this cation to CB7 and B^+^–CB7 differ the most (Table 2). In 4 μm B^+^ and CB7 solution, 1.6 mm Ca^2+^ or 5.6 mm K^+^ concentration is enough to outweigh the competitive binding of M^*n*+^ to CB7 by ternary‐complex formation. Above these concentrations B^+^ release from ternary complex B^+^–CB7–M^*n*+^ dominates over indirect decomplexation of B^+^–CB7 through competitive CB7–M^*n*+^ formation.

Association to form guest–CB7–M^*n*+^ ternary complexes has been suggested,^15^, ^18^–, 19c, 27 but neither their binding constants nor the effect of M^*n*+^ variation on the rate constants of the dissociation pathways has been revealed. Previous fluorescence‐lifetime measurements showed ternary‐complex formation of B^+^–CB7 with Na^+^ or 1‐butyl‐3‐methylimidazolium cation.19c Kinetic studies on the CB6 complex of 4‐methylbenzylammonium implied that not only competitive binding of K^+^ occurred, but also the ternary complex was produced.^27^ Association of cyclohexylmethylammonium–CB6 inclusion complex with Na^+^ was taken into account in the analysis of the salt‐concentration dependence of the apparent binding constants.^15^ The CB7 complex of the ditopic *N*‐phenyl‐2‐naphthylammonium was able to coordinate Na^+^ cation only if the phenyl moiety of the guest was embedded in the host cavity, and 51±2 s^−1^ was reported for the rate constant of guest release.^18^ This is about an order of magnitude larger than the corresponding *k*_out_(BCM) values found for B^+^–CB7–M^*n*+^ dissociation. In the latter case, the exit probably has a higher activation enthalpy. Previous studies demonstrated that the passage of B^+^ through the tight CB7 portal requires structural deformation of host and guest.^26^, ^28^ The release of the much smaller phenyl group is sterically less hindered and can occur without build‐up of steric/conformational strain.

### MDAP^2+^ inclusion into the CB7 cavity is competitively slowed down by CB7‐portal‐bound M^*n*+^ cations

**Figure 7:**
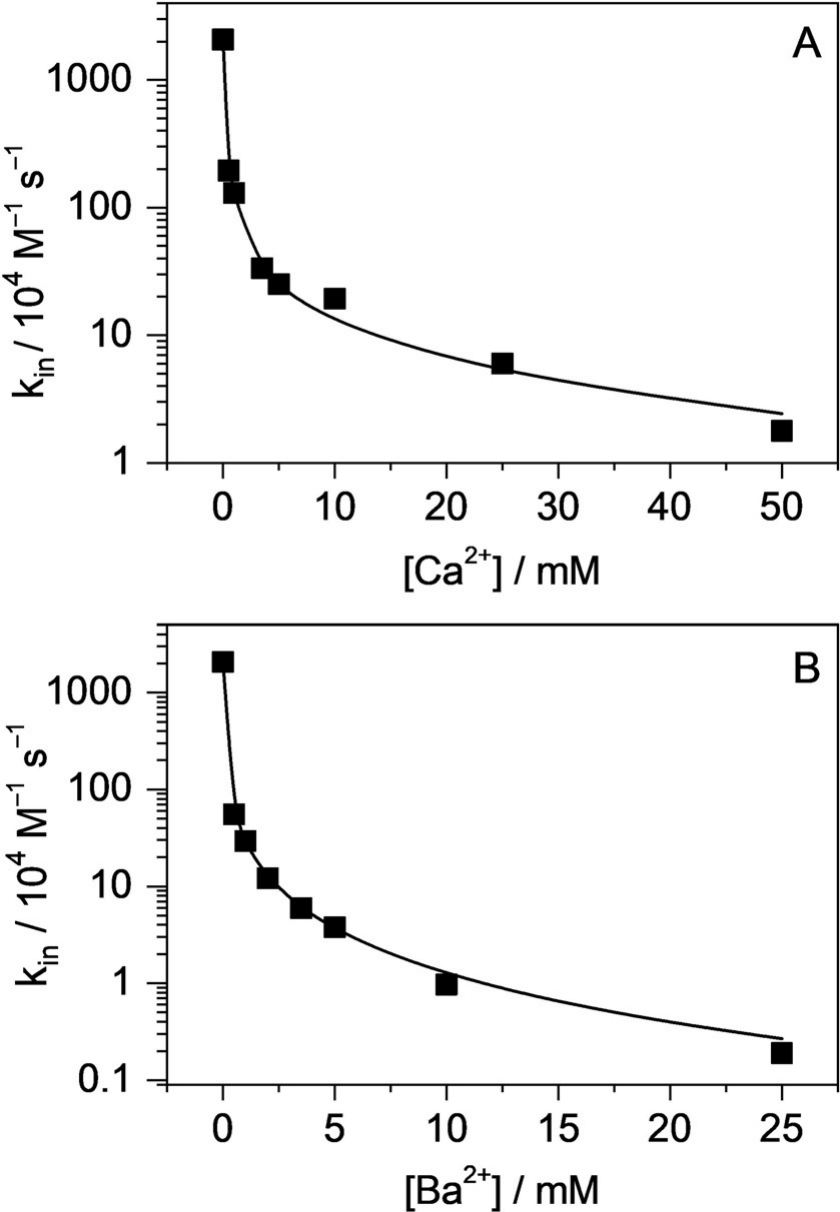
Rate constant of MDAP^2+^ ingression into CB7 as a function of A) Ca^2+^ and B) Ba^2+^ concentrations. The lines represent the fitting to Equation (2).

To reveal the salt effect on *k*_in_, equimolar solutions of MDAP^2+^ and CB7 (5 μm at *t*=0 s) were mixed and the rise of the fluorescence intensity was recorded at 454 nm at various Ca(NO_3_)_2_ or Ba(NO_3_)_2_ concentrations. A representative kinetic profile is shown in Figure S2 b in the Supporting Information. Figure 7 shows the considerable decrease of *k*_in_ with increasing salt concentration. The findings are attributed to lessening of the amount of unbound CB7 stemming from its competitive association with one or two M^*n*+^.

In general, M^*n*+^ cations associate with CB7 to form CB7–M^*n*+^ and (potentially also) M^*n*+^–CB7–M^*n*+^ complexes.^7^, ^17^, 20c These processes reduce the amount of free CB7 and consequently, decelerate the bimolecular guest ingression into CB7. (In a simple pictorial model, the bound cations can be considered to be lids closing the CB*n* portals.^9^, ^29^) If the guest–CB7–M^*n*+^ ternary complex is not produced and [CB7]≪[M^*n*+^], then the variation of *k*_in_ is expected to follow Equation (2):

(2) $$k_{\mathrm{in}}=k_{\mathrm{in}}^{0}\frac{1}{1+K_{\mathrm{CM}}\left[M^{n+}\right]+K_{\mathrm{CM}}K_{\mathrm{MCM}}{\left[M^{n+}\right]}^{2}}$$

where the rate constant of guest encapsulation in the absence of salt $k_{in}^{0}$ is multiplied by the fraction of free CB7 at total metal cation concentration [M^*n*+^] and *K*_CM_ and *K*_MCM_ are the equilibrium constants of CB7–M^*n*+^ and M^*n*+^–CB7–M^*n*+^ formation, respectively. The coordination of the second M^*n*+^ to CB7–M^*n*+^ has a small binding constant due to electrostatic repulsion (e.g., *K*_MCM_=11 m ^−1^ was reported for Na^+^ cation).19c Hence, M^*n*+^–CB7–M^*n*+^ is not expected to play an important role at the concentrations used in the determination of *k*_in_. Indeed, Equation (2) provides a good rationale for “mechanistically simple” MDAP^2+^ guest inclusion.

The best fits of the experimental results with Equation (2) provided *K*_CM_=17 000 m ^−1^ and *K*_MCM_=0 m ^−1^ for Ca^2+^, whereas 65 000 and 150 m ^−1^ were found for the binding constants of CB7–Ba^2+^ and Ba^2+^–CB7–Ba^2+^ production. The obtained *K*_MCM_ data can be considered estimates, because the quality of the fit is not sensitive to these values. The *K*_CM_ values are in accordance with the previously published results.^7^ The good agreement of the experimental data with the calculated curves in Figure 7 and the reasonable values of the derived parameters confirm that ternary complex MDAP^2+^–CB7–M^*n*+^ is not produced. The salt effect on the rate of MDAP^2+^ ingression into CB7 stems from the competitive binding of M^*n*+^ to the host.

### B^+^ inclusion occurs both into free CB7 and into the CB7–M^*n*+^ complex

**Figure 8:**
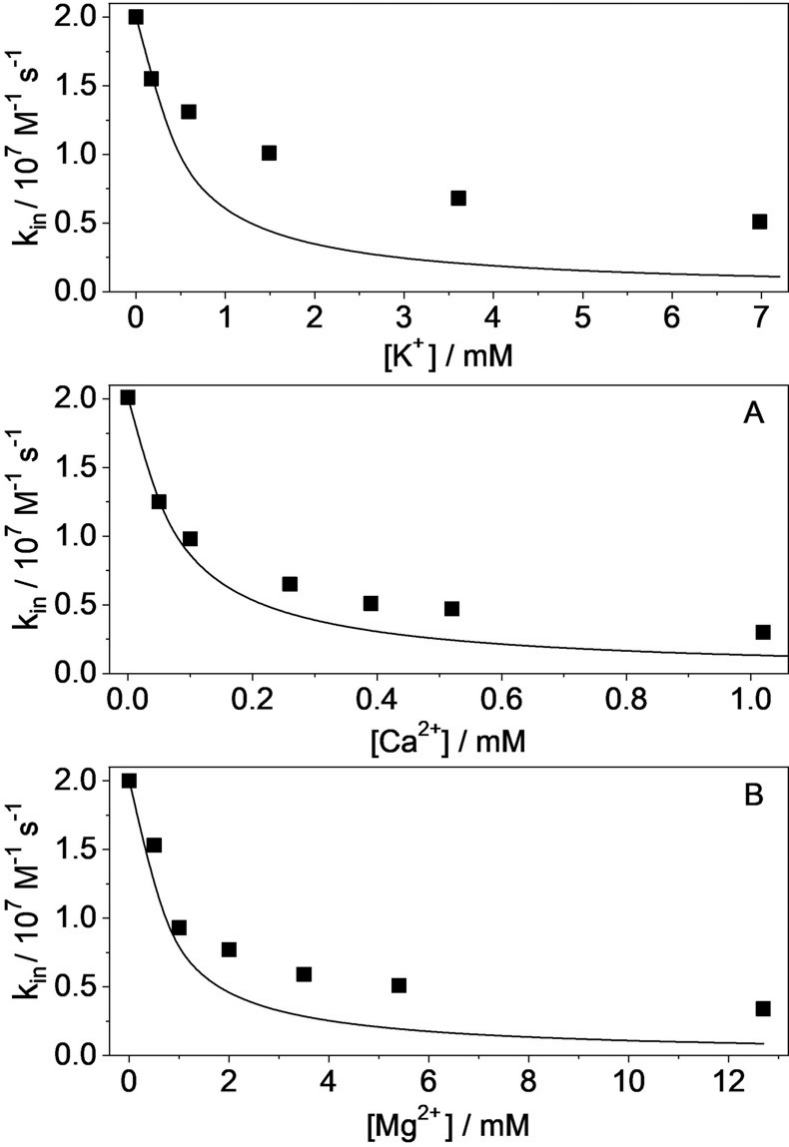
Comparison of the measured *k*_in_ rate constants (squares) with the values calculated if only competitive binding of M^*n*+^ to CB7 takes place and the B^+^–CB7–M^*n*+^ ternary complex is not produced (lines). The lines corresponds to *K*_CM_=2400, 14 000 and 1740 m ^−1^ for 1:1 association of K^+^, Ca^2+^ and Mg^2+^, respectively,^7^ and *K*_MCM_=0 m ^−1^.

To unravel how formation of the ternary complex B^+^–CB7–M^*n*+^ and the competitive binding of M^*n*+^ cations to CB7 modify the apparent rate constant of B^+^ encapsulation *k*_in_, fluorescence intensity versus time traces were recorded after rapid mixing of equimolar (0.25 or 0.5 μm at *t*=0 s) B^+^ and CB7 solutions in the presence of different amount of salts. Figure S3 in the Supporting Information shows representative results obtained in CaCl_2_ solutions. The initial slope of the signals decreases with increasing Ca^2+^ concentration, and this indicates deceleration of B^+^ capture. The concomitant lessening of the fluorescence intensity in the equilibrium arises from the combined effects of the diminution of the amount of B^+^–CB7 and the formation of the more weakly emitting B^+^–CB7–M^*n*+^ complex. The analysis of the kinetic results provided the *k*_in_ values presented in Figure 8 and Table S2 in the Supporting Information. For the system of CB7 and B^+^, substantial deviation of the experimental data from the trend predicted by Equation (2) is clearly found (see Figure 8), which points again to a “rich” complex formation/dissociation mechanism for this host–guest complex. From this, we can conclude that the salt effect on *k*_in_ cannot be rationalised by the simple competitive binding of M^*n*+^ to CB7. In line with the mechanistic pictures derived for B^+^ egression from CB7 and according to the principle of microscopic reversibility, the deviation between the experimentally determined ingression rates and the theoretical trends predicted by Equation (2) suggest that B^+^ enters not only free CB7 but also the cavity of CB7–M^*n*+^. The latter reaction has probably a smaller rate constant because the CB7‐bound M^*n*+^ sterically and electrostatically hinders the ingression of the cationic guest, but it cannot be ignored, because the rate and contribution of the process grows upon gradual addition of M^*n*+^ due to the rising CB7‐M^*n*+^ concentration.

## Conclusion

Selective measurement of the overall rate constant of inclusion‐complex dissociation *k*_out_ can shed light on subtle mechanistic details of the salt effect that would remain elusive in the generally conducted kinetic studies on guest ingression into a host. The separate determination of *k*_out_ is particularly beneficial because 1) it is not influenced by competitive association of the metal cations with CB7, 2) higher accuracy can be achieved, and 3) ternary guest–host–M^*n*+^ complex formation can be easily proved. The knowledge of *k*_out_ in a wide range of salt concentration is essential, not only to distinguish the bimolecular replacement of CB7‐bound guest by M^*n*+^ from the unimolecular guest release from guest–host–M^*n*+^, but also to discern the bimolecular expulsion of guest from such a ternary complex. The peculiar exit mechanism of B^+^ revealed in the present study may also be expected for neutral guests or for monocationic guests having highly delocalised charge and an extended aromatic ring system. The CB7 complex of such compounds may be prone to forming a ternary complex with metal cations. Voluminous, multiply charged cations exhibit higher affinity to inclusion complexes and expel guests more efficiently from the produced ternary complex in a bimolecular reaction. If salt‐concentration‐independent egression rate is favoured in an application of a CB7 complex, guests with multiple or localised charge may be a good choice. In fluorescence displacement assays, dilute host and guest solutions are preferable to minimise the effect of ternary complex production. The knowledge of the effect of metal cations on the kinetics of reversible host–guest binding may contribute to the rational design of salt‐responsive systems. The properties of the dynamic networks in supramolecular polymeric hydrogels^30^ could also be tuned by the addition of salts, and the control of the rate of host–guest association facilitates the adjustment of gelation kinetics.

## Experimental Section

Berberine chloride (B^+^Cl^−^, Sigma) was purified by chromatography on a silica gel (Merck) column with ethanol as eluent. 2,7‐Dimethyldiazapyrenium diiodide^23^ (MDAP^2+^2 I^−^) was synthesised from 1,3,6,8‐tetrahydro‐2,7‐dimethyl‐2,7‐diazapyrene by following the reported DDQ‐oxidation procedure.^31^ Metal salts and 1‐adamantylamine (Aldrich) were used as received. The latter compound is fully protonated at neutral pH because its conjugated acid has a p*K*_a_ value of 10.55. High‐purity CB7 was kindly provided by Dr. Anthony I. Day (University of New South Wales, Canberra, Australia). Alternatively, commercial CB7 can be desalinated by dialysis (Spectra/Por dialysis membrane, Biotech CE Tubing, MWCO 100–500 D, wet in 0.05 % sodium azide, norminal flat width 31 mm, diameter 20 mm, volume/length 3.1 mL cm^−1^, Part Number: 131060) Water was freshly distilled twice from dilute KMnO_4_ solution.

Stopped‐flow measurements of B^+^ release from CB7 were performed with an Applied Photophysics RX2000 rapid mixing unit connected to a Jobin‐Yvon Fluoromax‐P photon‐counting spectrofluorometer, whereas the binding kinetics of MDAP^2+^‐CB7 complex formation were studied with an SFA‐20 rapid kinetic accessory with a pneumatic drive unit from HI‐TECH Scientific connected to a Jasco FP‐8300 fluorescence spectrometer equipped with a 450 W xenon arc lamp, double‐grating excitation and emission monochromators. Fluorescence spectra were recorded with the same spectrometers without using the kinetic accessory. All measurements were carried out at 298 K.

The rate constant of guest release from the CB7 cavity *k*_out_ was determined by the previously reported method^22^ using the competitive strong binding of 1‐adamantylammonium cation (AH^+^) in CB7. Due to its ideal size complementarity and rigidity, AH^+^ has an extremely large association constant with CB7 (*K*=1.7×10^14^ m ^−1^), which guarantees its very slow exit from the CB7 cavity.^32^ Equimolar solutions of guest and CB7 were rapidly mixed in 1:1 volume ratio with AH^+^ solution in the presence of various salt concentrations. To ensure that the bimolecular inclusion was much faster for AH^+^ than for the guest, AH^+^ was employed in at least 20‐fold excess. As an indication of the negligible back‐formation of guest–CB7 complex after dissociation, we always checked that further increase of the [AH^+^]/[guest] molar ratio did not modify the derived *k*_out_ values. B^+^ release was examined at total concentrations of [B^+^]_T_=[CB7]_T_=0.25 μm and [AH^+^]_T_=5 μm at *t*=0 s. The excitation and monitoring occurred at 345 and 505 nm, respectively. MDAP^2+^ exit from CB7 was studied at [MDAP^2+^]_T_=[CB7]_T_=5 μm and [AH^+^]_T_=300 μm at *t*=0 s. The excitation and monitoring took place at 339 and 454 nm, respectively. The rate constants of ingression were measured by monitoring the fluorescence intensity change after 1:1 mixing of equimolar guest and CB7 solutions in the presence of various amounts of salts. Typical reactant concentrations at *t*=0 s were 0.5 and 5 μm. Fluorescence monitoring was carried out 505 and 454 nm for B^+^ and MDAP^2+^ inclusion, respectively. The experimental data were fitted to the numerical solution of a differential equation describing the time dependence of the fluorescence intensity to calculate the rate constant of inclusion *k*_in_ while keeping the separately determined *k*_out_ values constant.

## Conflict of interest

The authors declare no conflict of interest.

## Supporting information

As a service to our authors and readers, this journal provides supporting information supplied by the authors. Such materials are peer reviewed and may be re‐organized for online delivery, but are not copy‐edited or typeset. Technical support issues arising from supporting information (other than missing files) should be addressed to the authors.

Supplementary
