# Supplementary material for: Kinetics and Mechanism of Cation‐Induced Guest Release from Cucurbit[7]uril
Source: Chemistry. 2020 May 19;26(33):7433–41. doi: 10.1002/chem.201905633 (PMC7318709; doi:10.1002/chem.201905633)
Supplement: Supplementary file 1 — Supplementary [file CHEM-26-7433-s001.pdf]

Chemistry–A European Journal

Supporting Information

Kinetics and Mechanism of Cation-Induced Guest Release from Cucurbit[7]uril

Zsombor Miskolczy,[a] Mónika Megyesi,[a] László Biczók,*[a] Amrutha Prabodh,[b] and Frank Biedermann[b]

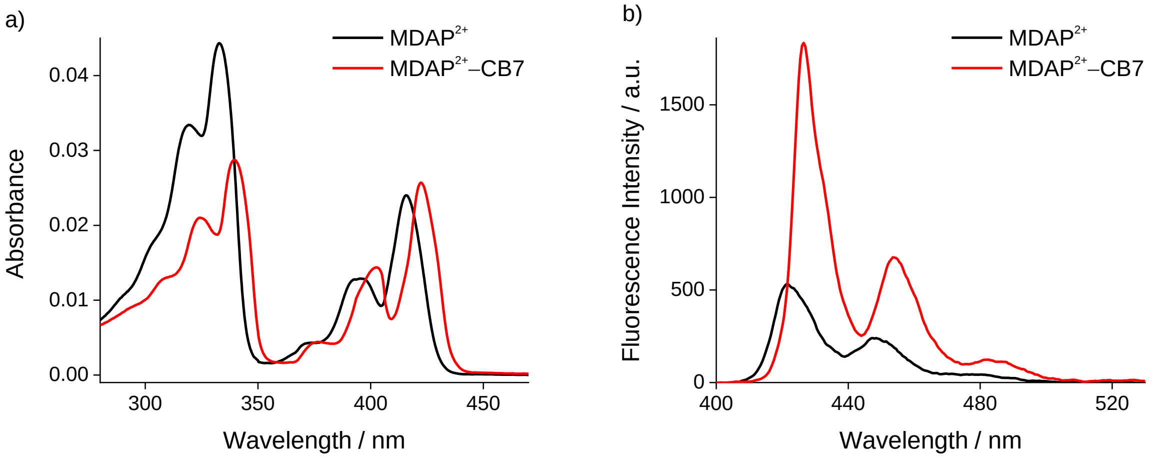

Fig. S1 (a) Absorption and (b) fluorescence spectra of 2 μM $MDAP^{2+}$ in the absence (black line) and in the presence of 8.6 μM CB7 (red line). Excitation occurred at 339 nm.

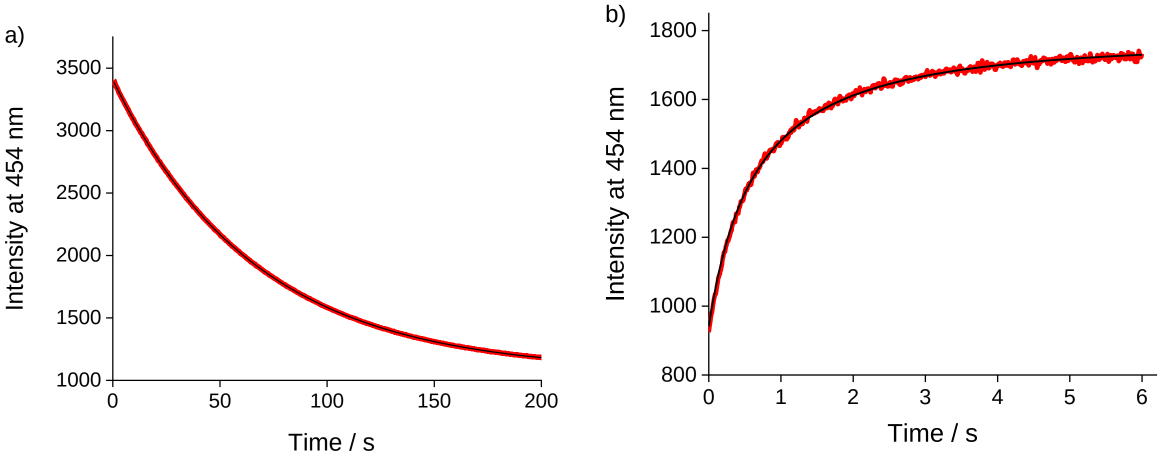

Fig. S2 (a) Stopped flow signals at 454 nm in $MDAP^{2+}$–CB7 solution (5 μM at t = 0 s) after mixing with the solution of AH^+ (300 μM at t = 0 s) in water. (b) Stopped flow signals at 454 nm after mixing $MDAP^{2+}$ and CB7 aqueous solutions. Initial concentrations at t = 0 s were 0.0708 μM. Excitation at 339 nm. The black lines represent the result of the nonlinear least-squares analysis.

Formula for the overall rate constant of B^+ exit from BC and BCM

The fluorescence intensity at the monitoring wavelength (*I(t)*) has contribution from BC and BCM emissions:

$$I(t) = \alpha[BC] + \beta[BCM] \qquad \text{(S1)}$$

Since the back formation of B^+–CB7 and B^+–CB7–M^{n+} is negligible in the presence of AH^+, the dissociation rates are defined as follows:

$$\frac{d[BC]}{dt} = -k_{out}(BC)[BC] \qquad \text{(S2)}$$

$$[BC] = [BC]_0 \exp(-k_{out}(BC)t) \qquad \text{(S3)}$$

$$\frac{d[BCM]}{dt} = -k_{out}(BCM)[BCM] \qquad \text{(S4)}$$

$$[BCM] = [BCM]_0 \exp(-k_{out}(BCM)t) \qquad \text{(S5)}$$

where $[BC]_0$ and $[BCM]_0$ represent the BC and BCM concentrations at t = 0 s

Substitution of eq. (S3) and (S5) into (S1) yields

$$I(t) = \alpha[BC]_0 \exp(-k_{out}(BC)t) + \beta[BCM]_0 \exp(-k_{out}(BCM)t) \qquad \text{(S6)}$$

Single exponential decay was observed with k_{out} apparent rate constant because $k_{out}(BC)$ and $k_{out}(BCM)$ do not differ sufficiently for the resolution of the two decay components.

$$I(t) = (\alpha[BC]_0 + \beta[BCM]_0)\exp(-k_{out}t) \qquad \text{(S7)}$$

The contribution of BC emissions to the overall fluorescence intensity:

$$I_{BC}(t)/I(t) = \alpha[BC]_0 \exp(-k_{out}t) / (\alpha[BC]_0 + \beta[BCM]_0)\exp(-k_{out}t)$$

$$I_{BC}(t)/I(t) = \alpha[BC]_0/(\alpha[BC]_0 + \beta[BCM]_0) \qquad \text{(S8)}$$

Similarly, the contribution of BCM emissions to the overall fluorescence intensity:

$$I_{BCM}(t)/I(t) = \beta[BCM]_0/(\alpha[BC]_0 + \beta[BCM]_0) \quad \text{(S9)}$$

The relationship between $[BCM]_0$ and $[BC]_0$ can be derived from the equilibrium constant of M^{n+} binding to BC:

$$[BCM]_0 = K_{BCM}[M]^{n+}[BC]_0 \quad \text{(S10)}$$

Substitution of (S10) into (S8) and (S9)

$$I_{BC}(t)/I(t) = \frac{\alpha}{\alpha + \beta K_{BCM}[M]^{n+}} = \frac{1}{1+\frac{\beta}{\alpha}K_{BCM}[M]^{n+}} \quad \text{(S11)}$$

$$I_{BCM}(t)/I(t) = \frac{\beta K_{BCM}[M]^{n+}}{\alpha + \beta K_{BCM}[M]^{n+}} = \frac{\frac{\beta}{\alpha}K_{BCM}[M]^{n+}}{1+\frac{\beta}{\alpha}K_{BCM}[M]^{n+}} \quad \text{(S12)}$$

The contributions of $k_{out}(BC)$ and $k_{out}(BCM)$ to the overall rate constant k_{out} are proportional to the contributions of BC and BCM emissions to the total fluorescence intensity.

$$k_{out} = k_{out}(BC)\frac{1}{1+\frac{\beta}{\alpha}K_{BCM}[M^{n+}]} + k_{out}(BCM)\frac{\frac{\beta}{\alpha}K_{BCM}[M^{n+}]}{1+\frac{\beta}{\alpha}K_{BCM}[M^{n+}]} \quad \text{(S13)}$$

Formulas for the calculation of B^+–CB7–M^{n+} / CB7–M^{n+} molar ratios and the concentrations of each component in equilibrium

As metal cations (M^{n+}) are in large excess relative to the other components, concentration of M^{n+} is not influenced by complex formations and practically equal to their total concentration (M).

B_T and C_T represent the total concentrations of B^+ and CB7, while B, C, BC, CM, and BCM denote the concentration of B^+, CB7, B^+–CB7, CB7–M^{n+} and B^+–CB7–M^{n+} , respectively.

M^{n+} + CB7 $\rightleftharpoons$ CB7–M^{n+} binding constant K_M

$$CM = K_M*C*M \quad (S14)$$

B^+ + CB7 $\rightleftharpoons$ B^+–CB7 binding constant K_B

$$BC = K_B*B*C \quad (S15)$$

B^+–CB7 + M^{n+} $\rightleftharpoons$ B^+–CB7–M^{n+} binding constant K_T

$$BCM = K_T*BC*M = K_T* K_B*B*C*M \quad (S16)$$

Material balance equations:

$$C_T = C + BC + BCM + CM \quad (S17)$$

$$B_T = B + BC + BCM \quad (S18)$$

From (S17) using (S14), (S15), and (S16)

$$C_T = C + K_B*B*C + K_T* K_B*B*C*M + K_M*M*C \quad (S19)$$

$$C = C_T / (1 + K_B*B + K_T* K_B*B*M + K_M*M) \quad (S20)$$

From (S18) using (S14), (S15), and (S16)

$$B_T = B + K_B*B*C + K_T* K_B*B*C*M \qquad (S21)$$

$$B + K_B*B*C + K_T* K_B*B*C*M – B_T =0 \qquad (S22)$$

From (S22) using (S20)

$$B + K_B*B*C_T / (1 + K_B*B + K_T* K_B*B*M + K_M*M) +$$

$$K_T* K_B*B*M*C_T / (1 + K_B*B + K_T* K_B*B*M + K_M*M) – B_T =0 \qquad (S23)$$

$$B*(1 + K_B*B + K_T* K_B*B*M + K_M*M) + K_B*B*C_T + K_T* K_B*B*M*C_T –$$

$$B_T*(1 + K_B*B + K_T* K_B*B*M + K_M*M) =0 \qquad (S24)$$

$$B^2*(K_B + K_T*K_B*M) + B*(1 + K_M*M + K_B*C_T + K_T*K_B*M*C_T – K_B*B_T –$$

$$K_T*K_B*B_T*M) – (1+K_M*M)*B_T = 0 \qquad (S25)$$

The solution of the second order equation (S25) gives B.

(S16) and (S14) provide

$$BCM / CM = K_T* K_B*B / K_M$$

C, CM, BC, and BCM can be calculated on the basis of (S20), (S14), (S15), and (S16), respectively.

Table S1 Calculated solution composition in equilibrium as a function of total Ca^{2+} concentration at 0.02 mM B^+ and 1 mM CB7 total concentrations [a]

$[Ca^{2+}]_{total}$ mM	$[B^+]$ 10^{-5} mM	$[B^+–CB7]$ 10^{-3} mM	$[B^+–CB7–Ca^{2+}]$ 10^{-3} mM.	[C] 10^{-3} mM	[CM] 10^{-3} mM
5	2.13	6.77	13.21	13.80	966.2
6	2.26	5.98	14.00	11.53	968.5
7	2.35	5.36	14.62	9.90	970.1
8	2.43	4.85	15.13	8.67	971.4
9	2.50	4.43	15.55	7.72	972.3
10	2.55	4.08	15.90	6.95	973.1
11	2.60	3.78	16.20	6.32	973.7
12	2.64	3.52	16.46	5.80	974.2

[a] Binding constants of B^+–CB7, B^+–CB7–Ca^{2+}, and CM formations are $K_{BC} = 2.3\times10^7$ M^{-1} (ref. 26), $K_{BCM} = 390$ M^{-1} (Table 1), and $K_{CM} = 14000$ M^{-1} (ref. 16), respectively.

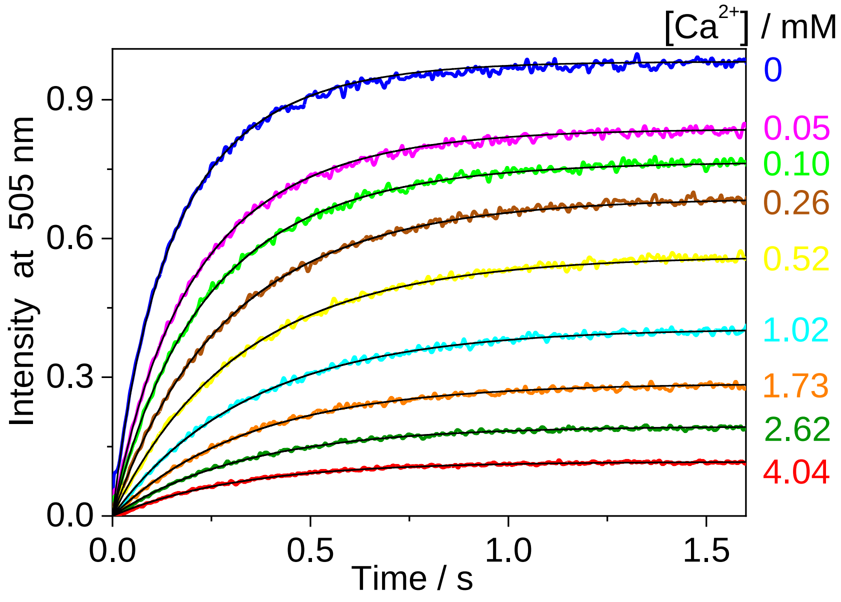

Fig. S3 Stopped-flow signals recorded after 1:1 mixing of equimolar (0.25 μM at t = 0 s) B^+ and CB7 solutions at various $CaCl_2$ concentrations. Excitation occurred at 345 nm.

Table S2 Effect of Li^+ and Ba^{2+} concentrations on the apparent rate constant of B^+ inclusion in CB7 (k_{in})

[LiCl] mM	k_{in} [a] 10^6 M^{-1} s^{-1}	[$BaCl_2$] mM	k_{in} [a] 10^6 M^{-1} s^{-1}
0	21.8	0	19.7
1.11	20.5	0.005	14.8
2.21	20.0	0.010	11.5
3.77	19.6	0.021	7.8
6.23	18.0	0.039	5.4
10.45	15.7	0.065	4.2
		0.098	4.0

[a] Estimated error ≈ 10 %
